# 

**Published:** 1842-05-14

**Authors:** 


### CONTENTS OF No. 20.

Dr. Reynell Coates' Contributions to Surgery,
Note on the means of avoiding Excoriations of the Heel and Perineum in Fractures of the Lower Extremities, treated by Permanent Extension, . . . . . . . . 305
Dr. E. Hartshorne's Clinical Reports,
Case of Severe Compound Fracture of both bones of the Leg, 308
EDITORIAL: Professor M'Clintock's Introductory Lecture, . . . . 312
Appointment of Dr. William Pepper, . . . . . . 312
ANALECTA: Syrup of Wild Cherry Bark, . . . . . . . 313
Syrup of Valerian, . . . . . . . . . 313
Dr. Liston on a Variety of False Aneurism, . : . 314
Dr. Griffin's Case of Complicated Fractures of both Thighs, and of the upper and lower Jaws, with Diastasis of the former, . . . . . . . . . . . 316
Dr. Charles Clay on the Treatment of Mesenteric Glandular Affections, . . . . . . . . . : 317
Death from Thomsonism, . . . . . . . . 320

